# Antenatal pelvic floor muscle exercise intervention led by midwives in England to reduce postnatal urinary incontinence: APPEAL feasibility and pilot randomised controlled cluster trial

**DOI:** 10.1136/bmjopen-2024-091248

**Published:** 2025-01-20

**Authors:** Christine MacArthur, Debra Bick, Victoria Salmon, Ellie Jones, Jean Hay-smith, Jonathan Bishop, Eleni Gkini, Karla Hemming, Sara Webb, Mark Pearson, Tim Coleman, Rohini Terry, Elizabeth Edwards, Helena Frawley, Eivor Oborn, Sarah Dean

**Affiliations:** 1 School of Health Sciences, University of Birmingham, Birmingham, UK; 2 Warwick Clinical Trials Unit, Warwick Medical School, University of Warwick, Coventry, UK; 3 University of Exeter Medical school, University of Exeter, Exeter, UK; 4 Rehabilitation Teaching and Research Unit, University of Otago, Wellington, New Zealand; 5 Birmingham Clinical Trials Unit, University of Birmingham, Birmingham, UK; 6 The Royal College of Midwives, London, UK; 7 Hull York Medical School, University of Hull, Hull, UK; 8 Division of General Practice, University of Nottingham, Nottingham, UK; 9 Birmingham Women’s and Children’s NHS Foundation Trust, Birmingham, UK; 10 Melbourne School of Health Sciences, The University of Melbourne, Melbourne, Victoria, Australia; 11 Warwick Business School, Warwick University, Coventry, UK

**Keywords:** Midwifery, OBSTETRICS, Pregnancy

## Abstract

**Objectives:**

To assess the feasibility of an intervention of midwifery support for antenatal pelvic floor muscle exercises (PFME) to prevent postnatal urinary incontinence (UI).

**Design:**

Feasibility and pilot cluster randomised controlled trial. Clusters were community midwifery teams.

**Setting:**

Community maternity antenatal care.

**Participants:**

One hundred seventy-five women; 186 midwives.

**Intervention:**

Midwifery training and resources for midwives and women to support antenatal PFME. Control clusters continued standard care.

**Outcomes:**

Women reporting: that their midwife explained how to do PFME, PFME adherence and postpartum UI. Midwives reporting: pre-post-training PFME confidence, intervention acceptability. Fidelity of training delivery and implementation.

**Results:**

Ninety-five midwives in intervention clusters; 91 midwives in control clusters. Of 998 women sent questionnaires, 175 responded: 15.8% in intervention, 16.4% in control clusters. Women’s characteristics in both trial arms were similar and characteristics of respondents and non-respondents were similar. Sixty-five percent (95% CI 56.9% to 72.4%) of women in intervention clusters reported their midwife explained how to do PFME vs 38% (95% CI 24.6% to 51.2%) in control clusters. Fifty percent (95% CI 24.1% to 77.1%) of women in intervention clusters vs 38% (95% CI 12.4% to 67.1%) in control clusters reported doing enough PFME to potentially prevent UI. Fourty-four percent (95% CI 32.0% to 56.1%) of women in intervention clusters reported UI vs 54% (95% CI 42.2% to 65.8%) in control clusters.

Intervention training was delivered with fidelity and received positively. Midwives reported improvements in PFME confidence/knowledge (median increase of at least 1 point on a 0–4 scale for each of eight questions). Midwives (26%) most frequently reported insufficient time as an implementation barrier.

**Conclusions:**

This pilot trial produced consistent new findings that training and resourcing midwives to teach and support pregnant women to undertake PFME is acceptable and feasible for women and midwives. It increased the number of women who are informed about PFME, with potential to improve PFME adherence and reduce postpartum UI. Recent changes to the National Health Service perinatal pelvic healthcare means a full trial is not possible.

**Trial registration number:**

ISRCTN10833250.

**STRENGTHS AND LIMITATIONS OF THIS STUDY**

- Service level cluster design of this feasibility and pilot randomised controlled trial reduced sources of bias commonly associated with pelvic floor muscle exercises trials: women were blind to trial arm during both receipt of the intervention/control and when reporting their outcomes.
- Extensive Patient and Public Involvement and Engagement and Stakeholder activities throughout the trial strengthened research and intervention design.
- Online questionnaires, which would likely have improved return rates, were not able to be sent to women because of data protection issues. However, aggregate data were available for all women who delivered in the sample month and did not return a questionnaire, showing most characteristics were similar.

## Introduction

 Urinary incontinence (UI) in women is common and pregnancy and birth are the main risk factors. A systematic review showed 33% of women experienced UI in the first 3 months after childbirth^1^; a cohort study showed over 40% experienced UI sometime in the year after childbirth.^2^ Another cohort study showed two-thirds to three-quarters of women who had postpartum UI still experienced it 12 years after birth^3^; and at 26 year follow-up of this cohort overall UI prevalence was 61%.^4^ UI places a large burden on women’s health and quality of life^5^ and substantial pressure on health services and wider societal costs.^6^

There is clear evidence in a Cochrane review^7^ that antenatal pelvic floor muscle exercises (PFME) are effective in decreasing the likelihood of postnatal UI. In prevention trials, pregnant women without prior UI randomised to supervised PFME training were 29% (RR 0.71, 95% CI 0.54 to 0.95) less likely to report UI up to 6 months postpartum than women randomised to no PFME/usual antenatal care. A more recent systematic review has shown similar findings.^8^ Most interventions in the previous trials were undertaken by specialist health professionals, mainly physiotherapists, but in the UK it is only midwives who routinely see all women during pregnancy. Thus, it was considered important to investigate whether midwives could incorporate a suitable PFME intervention into their routine antenatal care; and whether this might result in women undertaking appropriate PFME frequently enough to prevent postnatal UI.

The Antenatal Preventative Pelvic Floor Exercises and Localisation (APPEAL) research programme comprised a set of interlinked studies with the overarching aim of improving implementation of antenatal PFME to reduce the likelihood of UI in women following childbirth. Its first objectives were to investigate current antenatal care in relation to PFME support for women from midwives in a critical interpretative synthesis literature review and an ethnographic study.^9 10^ Findings showed that it was unrealistic to expect women and non-specialist health professionals to implement PFME without reforming policy and service delivery to genuinely support them in this endeavour^11^; and that women and midwives know PFME training is important, but often midwives do not communicate to women the benefits available from PFME.^10^ There was widespread lack of confidence among women and midwives about initiating conversations about PFME and UI, exacerbated by misunderstandings and lack of clear guidelines and policy.^10^

Following these studies, the APPEAL team worked with women, midwives and a wide range of stakeholders to develop an intervention designed to increase the likelihood of midwives teaching and supporting women to do PFME during pregnancy. This study reports the feasibility and pilot cluster randomised controlled trial (RCT) to investigate this intervention. APPEAL was funded by the National Institute for Health Research Programme Grant (RP-PG-0514–20 0 02).

## Methods

A feasibility and pilot cluster RCT was undertaken to evaluate the intervention using an outcome questionnaire sent to women; together with process evaluation. Feasibility was defined as the process of evaluating whether a trial is possible to conduct and if all the necessary components work together. The full protocol was registered and published^12^ and a long and shorter version of the outcome questionnaire was tested to consider the effect of length on response rate, showing that a shorter version did not improve response.^13^

### Clusters

Community midwifery teams (clusters) at two National Health Service (NHS) units in the West Midlands of England were randomly allocated in 1:1 ratio to intervention or control. A minimisation algorithm ensured approximate balance over the variables: midwifery team size (defined by average number of monthly births) and NHS unit. Midwives in teams allocated to the intervention received APPEAL PFME training. Midwives in control teams continued with standard antenatal care. Blinding of midwives providing care was not possible, but the cluster design (using community midwifery teams) meant women receiving antenatal care were unaware of whether their midwife had received APPEAL training or not hence reported their outcomes blind to their trial arm allocation. Due to the nature of the outcome data being about whether the PFME advice, explanation and information pack were given by the midwife, those responsible for conducting trial analyses could not be blind to allocation.

### Intervention

The intervention comprised a 90 min training session (delivered online due to COVID-19 restrictions at the time), led and facilitated by two trial research midwives. Following training, intervention midwives were asked to incorporate PFME advice, explain how to do PFME and support all pregnant women in their care with this. They introduced the topic of pelvic floor health at antenatal ‘booking’ appointment or as soon as possible after. Midwives gave women an APPEAL resource pack, including an APPEAL leaflet with PFME information, APPEAL logo stickers to use as reminders and links to the recommended apps (evaluated by a PPIE group during development). Midwives asked women at all subsequent antenatal appointments about PFME progress and any problems with PFME or incontinence. Each team chose a PFME champion midwife who received additional training to provide ongoing support to the team. In some teams, specifically where a high proportion of women were non-English speaking, Maternity Support Workers who provided translation, were also trained.

### Study population and data collection

The intervention training period was from January to March 2021. Women who gave birth during December 2021 were sent the postal questionnaire at 10–12 weeks postpartum; this cohort was the sample group to assess quantitative trial outcomes. Because of data protection issues we were not allowed to send online questionnaires. Choosing this month meant women giving birth had all their antenatal care provided after midwives in the intervention clusters had received their APPEAL training. Exclusions to being sent a questionnaire were having had stillbirth/neonatal death, severe mental health problems or infants taken into care, providing these data were available in maternity records. One reminder was sent if no questionnaire was returned after 2 weeks. The questionnaires, with accompanying information sheets, were sent by research midwives employed at each NHS site to all women who had given birth at the site during the sample month. This included women who were ‘out-of-area’ which means that they had not had antenatal care from any of the midwifery teams in the trial and women who had no midwifery team recorded in case notes. At the stage of sending the questionnaires, it had not been possible to remove these women from the sample. Questionnaires were returned to participating NHS Trusts and transferred securely in line with standard data protection procedures to the Birmingham Clinical Trials Unit (BCTU) for data cleaning and analysis by BCTU statisticians.

Returned questionnaire data were linked to women’s demographic, obstetric and infant data which were obtained (on consent when questionnaire was returned) from hospital records by the NHS midwives. Similar data from hospital records on all women who gave birth during the same month but who did not return a questionnaire were obtained to summarise characteristics of respondents and non-respondents. However, because of data protection these data could only be provided to the researchers in anonymised aggregate format. This allowed women who had responded to be separated from respondents who were out-of-area, but it was not possible to remove the out-of-area and no recorded midwife team women from the non-respondent group.

The women’s questionnaire included questions about PFME information received from their midwife, their practice of PFME and UI symptoms (online supplemental material 1). Main pre-specified pilot trial outcomes included: whether the intervention was implemented as planned, with assessment based on whether women reported that their midwife had explained how to perform PFME during pregnancy; women’s adherence to PFME was assessed by whether they had undertaken PFME during pregnancy a few times a week or more (considered frequent enough to prevent UI). UI was ascertained using the International Continence on Incontinence Questionnaire—Urinary Incontinence Short Form (ICIQ-UI SF).^14^ Urine leakage at the start of pregnancy, which is a major risk factor for postpartum UI^15^ was also assessed in the women’s questionnaire. Faecal incontinence (FI) at 10–12 weeks was assessed using the Revised Faecal Incontinence Scale (RFIS)^16^ but was not a major focus of the study as the Cochrane review had not shown the effectiveness of PFME for this outcome.^7^

#### Process evaluation

Process evaluation had three data collection phases. The first phase involved collecting demographic and work experience details of the midwives attending the training and before-and-after training session evaluation using Likert scale questions and free text data were used to assess midwife knowledge and confidence in teaching PFME and to understand training acceptability; and observations of training using checklists to assess fidelity of training delivery were conducted. The second phase was during the intervention period when online audio-recorded interviews were conducted with midwives to assess the acceptability of implementing APPEAL; and towards the end of the intervention period one survey of midwives to assess whether the various elements of APPEAL were undertaken. The third phase occurred after women had completed trial outcomes, women and midwives from both trial arms were interviewed to further explore acceptability and possible contamination. All women who gave consent to be contacted for an interview when they returned the postnatal questionnaire were approached via phone about taking part in an interview. A similar process was used to approach midwives about taking part in interviews, they were sampled from across the clusters after indicating on their demographic/work experience questionnaire that they consented to being approached for an interview.

All interviews were conducted by experienced female qualitative researchers who did not have prior relationship with participants via online video conferencing platforms. Interviewers introduced themselves as members of the research team but did not give further information regarding any professional qualifications (such as being a qualified midwife or physiotherapist). Topic guides (onlinesupplemental materials 2^6^) for the various midwives’ and women’s interviews were developed based on previous work packages including: critical interpretive synthesis,^11^ focused ethnographic observations of clinical practice^10^ and input from Patient and Public Involvement and Engagement (PPIE) advisory groups.

### Analyses

An important aim of the feasibility and pilot trial was to test whether a definitive trial would be possible, therefore the sample size was based on number and size of clusters needed to estimate return rate of questionnaires (across trial arms) to an acceptable level of precision. The overall sample size target was around 1400 (17 clusters, average size 82) to estimate 95% CI for return rate to maximum width of 17.2%.

Analyses were based on intention-to-treat principle. Data analysis was descriptive and mainly focused on CI estimation, with no hypothesis testing. Analysis methods included:

- Continuous endpoints summarised using means and SD, by arm.
- Categorical (dichotomous) endpoints (eg, adherence to PFME). Number of participants and percentages experiencing the event were summarised by arm.

For total scores and dichotomous feasibility outcomes, summary measures and 95% CIs per trial arm were estimated using cluster-level analysis based on t-distributions with K-1 df (K denotes number of clusters per group) and transformation where necessary (and weighting if variation in cluster sizes).

As the process evaluation analysis objectives related to feasibility and acceptability of the training, fidelity of training delivery and implementation, and any between-group contamination; the qualitative analysis did not aim for data saturation but rather sufficient information power to address objectives. Checklist data, free text data and any field notes made by researchers during interviews were analysed with content analysis or summarised descriptively. Thematic analysis was used for transcribed interview data by two qualitative researchers using NVivo software.

### Patient public involvement and engagement

Patients and public were involved throughout the programme of work using multiple methods. Two patient representatives were part of the research management team and a PPIE advisory group included six mothers with young children, with nine meetings held in the community. The PPIE advisors designed the APPEAL logo, co-developed the intervention and resources for women, helped refine format and content of the intervention package in the earlier programme phases and supported with interpretation of key study findings. Study findings were disseminated to key stakeholders, including PPIE contributors, at a celebration event.

## Results

**Figure 1:**
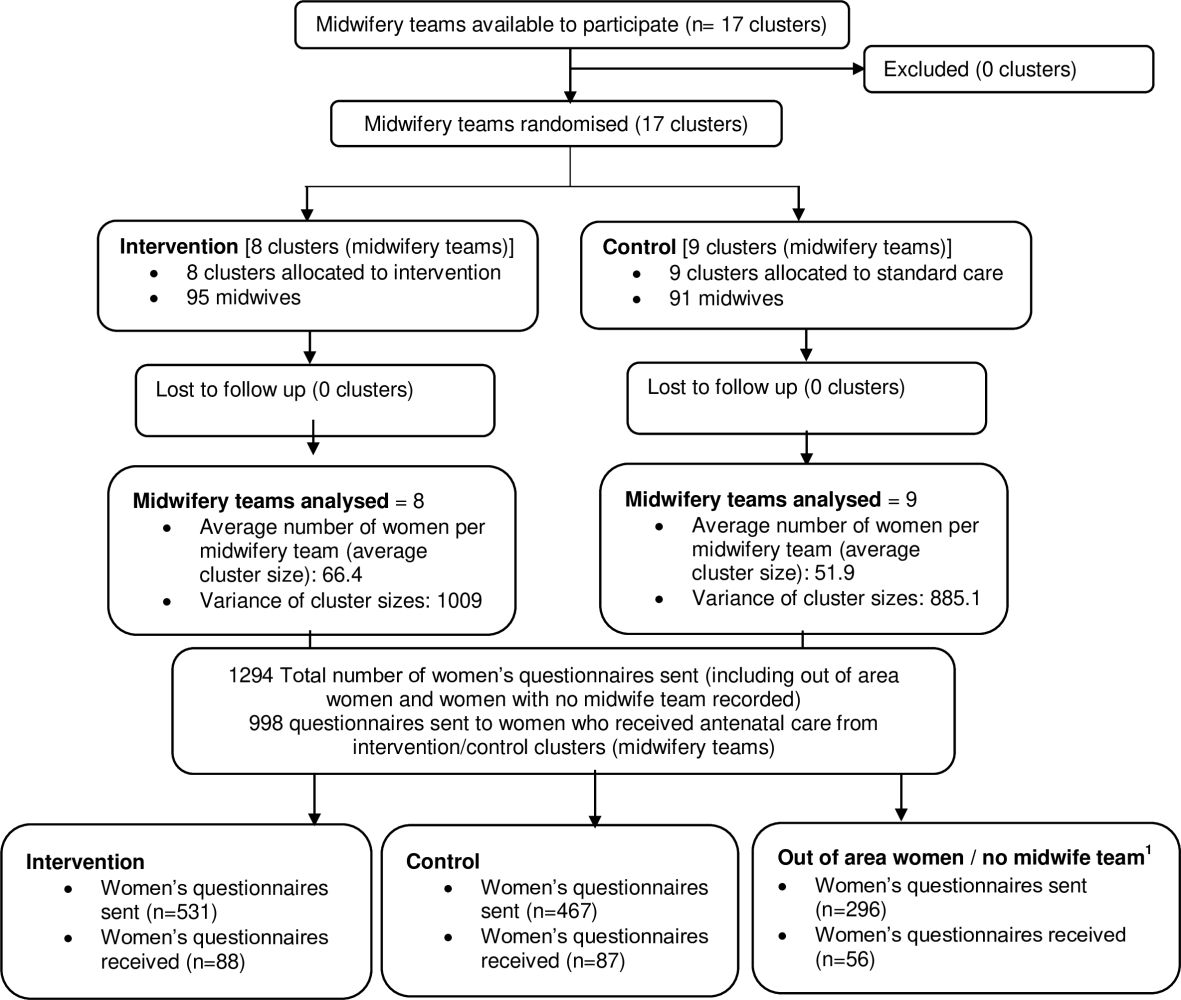
CONSORT (The Consolidated Standards of Reporting Trials) flow diagram. ^1^Women who gave birth in the hospital but had not received antenatal care from trial community midwife team clusters—out-of-area women or women who did not have a midwife team recorded in their case notes.

All 17 midwifery teams (clusters) across both participating NHS trusts were randomised (8 to intervention and 9 to control), comprising 186 midwives (95 intervention and 91 control). A total of 1294 women were sent a postpartum postal questionnaire (998 of whom were women known to have received care from a midwifery team in the trial) and 175 (17.5%, 95% CI 11.6% to 21.4%) were returned: 88/531 (16.6%) in intervention clusters, and 87/467 (18.6%) in control clusters (figure 1). A further 56 women who lived out-of-area (n=26) or had no midwife team recorded in case notes (n=30) returned questionnaires but had to be excluded as they had not received antenatal care from midwives known to be involved in the trial. All women who gave birth during the sample month were included in the group of women who did not return questionnaires (n=1063) in comparing baseline characteristics (table 1) and was based on aggregate case-note data.

**Table 1:** Demographic, obstetric and infant baseline characteristics by groups and overall

	Women who returned questionnaire from trial teams (respondents)			Women who did not return questionnaire (non-respondents)*
	Intervention(n=88)	Control(n=87)	Overall(n=175)	(n=1063)
**Demographic**				
Age, years				
Mean (SD)	31.6 (4.9)	31.9 (5.5)	31.8 (5.2)	29.6 (NA†)
Missing	10	10	20	0
Ethnicity				
British	40 (59.7%)	37 (62.7%)	77 (61.1%)	371 (43.2%)
Irish	1 (1.5%)	0 (0%)	1 (0.8%)	5 (0.6%)
White Other	5 (7.5%)	1 (1.7%)	6 (4.8%)	46 (5.4%)
White and Black African/Caribbean	0 (0%)	0 (0%)	0 (0%)	15 (1.7%)
White and Asian/Mixed Other	0 (0%)	0 (0%)	0 (0%)	13 (1.5%)
Indian	0 (0%)	3 (5.1%)	3 (2.4%)	33 (3.8%)
Pakistani	3 (4.5%)	7 (11.9%)	10 (7.9%)	205 (23.9%)
Bangladeshi	2 (3.0%)	0 (0%)	2 (1.6%)	16 (1.9%)
Asian other	6 (9.0%)	7 (11.9%)	13 (10.3%)	29 (3.4%)
Black African/Caribbean/other	4 (6.0%)	3 (5.1%)	7 (5.6%)	63 (7.3%)
Any other ethnic group	6 (9.0%)	1 (1.7%)	7 (5.6%)	62 (7.2%)
Missing	21	28	49	205
Parity				
Nulliparous	34 (45.3%)	28 (36.8%)	62 (41.1%)	367 (34.5%)
Multiparous	41 (54.7%)	48 (63.2%)	89 (58.9%)	696 (65.5%)
Missing	13	11	24	0
**Obstetric**				
Onset of labour				
Spontaneous	42 (55.3%)	39 (50.0%)	81 (52.6%)	429 (41.7%)
Induced	12 (15.8%)	23 (29.5%)	35 (22.7%)	307 (29.8%)
N/A-elective C section	22 (28.9%)	16 (20.5%)	38 (24.7%)	293 (28.5%)
Missing	12	9	21	34
Mode of birth				
Ventouse	10 (13.3%)	11 (14.1%)	21 (13.7%)	61 (5.7%)
Forceps	4 (5.3%)	4 (5.1%)	8 (5.2%)	49 (4.6%)
Caesarean section	26 (34.7%)	24 (30.8%)	50 (32.7%)	405 (38.1%)
Spontaneous vaginal birth	35 (46.7%)	39 (50.0%)	74 (48.4%)	547 (51.5%)
Missing	13	9	22	1
Anaesthetic				
Spinal	23 (79.3%)	23 (76.7%)	46 (78.0%)	345 (69.4%)
Epidural	5 (17.2%)	7 (23.3%)	12 (20.3%)	122 (24.5%)
General	1 (3.5%)	0 (0%)	1 (1.7%)	30 (6.0%)
Missing	59	57	116	566
Analgesia				
Yes	39 (72.2%)	50 (80.7%)	89 (76.7%)	524 (49.3%)
No	15 (27.8%)	12 (19.3%)	27 (23.3%)	539 (50.7%)
Missing	34	25	59	0
Perineal trauma				
First degree	6 (8.3%)	7 (9.1%)	13 (8.7%)	86 (15.2%)
Second degree	21 (29.2%)	27 (35.1%)	48 (32.2%)	223 (39.5%)
Third/fourth degree	3 (4.2%)	1 (1.3%)	4 (2.7%)	8 (1.4%)
None	42 (58.3%)	42 (54.5%)	84 (56.4%)	248 (43.9%)
Missing	16	10	26	498
Episiotomy				
Yes	17 (23.3%)	16 (21.1%)	33 (22.2%)	143 (24.0%)
No	56 (76.7%)	60 (78.9%)	116 (77.8%)	453 (76.0%)
Missing	15	11	26	467
Duration of second stage (min)				
Median (IQR)	35 (2,143)	36 (10,77)	36 (8,94)	NA†
Missing	62	50	112	254
**Infants**				
Gestation at birth (weeks)				
Mean (SD)	39.6 (1.6)	39.3 (2.2)	39.4 (1.9)	36.0 (NA†)
Missing	10	9	19	0
Birth weight (g)				
Mean (SD)	3356 (435)	3325 (499)	3340 (467)	3171 (NA†)
Missing	10	9	19	0
Head circumference (cm)				
Mean (SD)	34.5 (1.3)	34.2 (1.2)	34.4 (1.2)	33.3 (NA†)
Missing	15	13	28	48

* 1294 women sent a questionnaire, and 1063 did not return one. The respondent group excludes out-of-area women who sent back a questionnaire, but the non-respondent group includes out-of-area and no recorded midwife team women who did not return a questionnaire because this data had to be in aggregate format.

† In case notes. Trust could not supply figure.

Demographic, obstetric and infant characteristics of the women were shown to be similar across both trial arms (table 1) as were the proportions of women reporting no urine leakage at the start of pregnancy (68% intervention and 70% control).

Comparisons of demographic, obstetric and infant characteristics among the women who did and did not return questionnaires showed that most characteristics were similar. Data suggested some differences in proportions of women from ethnic minority groups (fewer among respondents) and parity (fewer multipara among respondents) (table 1).

Table 2 shows the data reported by women in relation to their midwife PFME support during pregnancy and their practice of PFME. In the intervention clusters, 65% (95% CI 56.9% to 72.4%) of the women said their midwife explained to them how to do PFME, relative to 38% (95% CI 24.6% to 51.2%) of women in the control clusters. The prespecified assessment of adherence to PFME was whether women reported having undertaken PFME a few times a week or more and 50% (95% CI 24.1% to 77.1%) of the women in the intervention clusters and 38% (95% CI, 12.4% to 67.1%) in control clusters reported this adherence.

**Table 2:** Women’s report of midwife support for pelvic floor muscle exercises (PFME) in pregnancy, women’s pelvic floor muscle exercises practice, and UI prevalence

Outcomes from questionnaire	Response	Intervention (n=88)	Control(n=87)
Key pilot outcomes			
Did your midwife explain how to perform PFME when you were pregnant?	Yes	57 (64.8%)	33 (37.9%)
	95% CI	56.9 to 72.4*	24.6 to 51.2†
Women’s pre-defined adherence in performing PFME in pregnancy‡	Yes	43 (50.0%)	33 (38.4%)
	Missing	2	1
	95% CI	24.1%–77.1%§	12.4%–67.1%¶
Prevalence of UI**	Yes	39 (44.3%)	47 (54.0%)
	95% CI	32.0% to 56.1%††	42.2% to 65.8%‡‡
Further outcomes			
Did your midwife advise you to perform PFME when you were pregnant?	Yes	73 (83.0%)	54 (62.1%)
Did your midwife give you a pack of information on PFME when you were pregnant?	Yes	49 (57.7%)	17 (19.8%)
	Missing	3	1
When did your midwife give you the pack of information on PFME?	Never	35 (42.7%)	65 (77.4%)
	At first (booking) appointment	15 (18.3%)	10 (11.9%)
	At second antenatal appointment	15 (18.3%)	3 (3.6%)
	At later antenatal appointment	17 (20.7%)	6 (7.1%)
	Missing	6	3
How often did your midwife talk to you about PFME when you were pregnant?	Never	13 (14.8%)	30 (34.5%)
	Only at booking appointment	20 (22.7%)	15 (17.2%)
	Occasionally	33 (37.5%)	22 (25.3%)
	Every antenatal appointment	18 (20.5%)	10 (11.5%)
	Can't remember	4 (4.5%)	10 (11.5%)
Did your midwife ever ask you if you had any difficulties with performing PFME?	Yes	27 (31.0%)	8 (9.3%)
	Missing	1	1
Before you were pregnant, have you ever been taught or learnt how to perform PFME?	Yes	36 (41.4%)	39 (45.4%)
	Missing	1	1
How often did you perform PFME when you were pregnant?	Never-not advised	9 (10.2%)	18 (20.7%)
	Never- other reasons	9 (10.2%)	4 (4.6%)
	Few times a month	19 (21.7%)	27 (31.0%)
	Once a week	6 (6.8%)	4 (4.6%)
	Few times a week	23 (26.1%)	17 (19.5%)
	Once a day	11 (12.5%)	5 (5.8%)
	Few times a day	9 (10.2%)	11 (12.6%)
	Can't remember	2 (2.3%)	1 (1.2%)
Do you currently perform PFME?	Yes	46 (66.7%)	45 (64.3%)
	Missing	19	17
How often did you do PFME over the last month?	Never-not advised	9 (10.2%)	12 (13.8%)
	Never-other reasons	10 (11.4%)	13 (14.9%)
	Few times a month	25 (28.4%)	23 (26.4%)
	Once a week	5 (5.7%)	4 (4.6%)
	Few times a week	24 (27.3%)	17 (19.5%)
	Once a day	6 (6.8%)	9 (10.4%)
	Few times a day	9 (10.2%)	(10.4%)

* 95% CI CI around the proportion who responded ‘Yes’, estimated using t distribution with 7 degrees of freedom (df).

† 95% CI CI around the proportion who responded ‘Yes’, estimated using t distribution with 6 df.

‡ Women’s self-reported adherence in performing antenatal PFME was assessed by response to the question: ‘How often did you perform PFME when you were pregnant?’ and a binary outcome defined as YES if answer was FEW TIMES A WEEK, ONCE A DAY, or FEW TIMES A DAY and NO if answer was: NEVER, WAS NEVER ADVISED, NEVER, OTHER REASONS, FEW TIMES A MONTH, or ONCE A WEEK. If the answer was CAN’T REMEMBER, or missing then binary outcome was missing.

§ 95% CI CI around the proportion who responded ‘Yes’, estimated after natural log transforming the data using cluster-level analysis, t distribution with df=7, and weighted by the cluster size.

¶ 95% CI CI around the proportion who responded ‘Yes’, estimated after natural log transforming the data using cluster-level analysis, t distribution with df=6, and weighted by the cluster size.

** UI prevalence was determined by the question: How often do you leak urine? and defined as NO if the answer was ‘never’ and YES if any other response.

†† 95% CI CI around the proportion who responded ‘yes’ estimated using a t distribution with 7seven df.

‡‡ 95% CI CI around the proportion who responded ‘yes’ estimated using a t distribution with 6six df.

PFMEpelvic floor muscle exercises

Among the women in the intervention clusters, 44% (95% CI 32.0% to 56.1%) reported UI and 54% (95% CI 42.2% to 65.8%) reported UI in the control clusters. Eighteen percent (95% CI 6.6% to 28.9%) of women reported FI in the intervention clusters and 13% (95% CI 4.8% to 21.2%) in control clusters.

### Process outcomes

Training fidelity was checked by observing the number of key statements delivered relative to those listed in the trainer’s manual which provided the key statements as a speaker script. During the initial training session, 75.8% of statements were delivered, which increased to 92.4% in two subsequent sessions. All 95 midwives in the intervention cluster teams received training, with those on sick or maternity leave trained on return to work. All teams appointed a midwife champion. Midwives showed clear increases in confidence about all aspects of PFME after training, with median increase of at least 1 point (0–4 scale) for each of eight questions.

Eighteen intervention midwives were approached for interview during the trial implementation period, with the aim to sample across the clusters, and to include team leads and champions. Thirteen in-depth interviews (average duration 40 min) were conducted including three team leads and four champions. Findings indicated positive responses about the training ‘I’m enthusiastic about it’; ‘it’s a very good fit’ with personal and professional values and important to women; training was perceived to be effective ‘I think it should help get that message across’; the intervention supported personal self-efficacy ‘I do feel confident’; the training made sense ‘I’ve never thought about the impact of pregnancy on the pelvic floor (before the training)’. There was some ambivalence mainly due to workload, limited time in antenatal appointments and system pressures: the burden of implementation ‘it just feels a bit impossible’; remembering everything; and opportunity costs ‘so many other priorities’; plus, a sense of hopefulness rather than certainty regarding effectiveness, with concerns they would not always be able to put the training into practice or that women would not do PFME. Interviews with team champions showed they found team meetings useful for peer support. Interviews with intervention midwives revealed benefits of a champion for support and advice.

At the end of the intervention period, an implementation evaluation questionnaire was completed by intervention midwives (n=59, 62% of those remaining in teams) (online supplemental material 7). They reported which elements of APPEAL they delivered to most/all of the women. The top three were: raising the topic (88%), giving the resource bag (68%) and explaining how to do a PFME contraction (68%). The least frequently delivered was practising a contraction in antenatal clinic (45%). In free text, respondents reported resource bags (n=31), prompt cards (n=17) and team champions (n=16) as the most important resources to support implementation. The top three most reported barriers to implementation were: ‘lack of time’ (n=39); ‘forgetting’ (n=29) and ‘language barriers’ (n=26).

Fifty-three intervention midwives and n=24 control midwives who had agreed to be contacted were approached to take part in post-trial interviews. Of these, n=22 intervention midwives and n=24 control midwives responded to telephone contact. Reasons given for not taking part in interviews included being off work, moving to a new team, not interested, being too busy, maternity leave or retiring from midwifery. Midwives from the intervention group (n=6) gave in-depth interviews (average duration 38 min) revealing positive experiences of training but some reported increased implementation inconsistency over time. Suggested opportunities for improving implementation included: longer appointments; prompts on maternity records; training updates; greater accessibility of women’s resources (eg, online leaflets); and more understanding of referral processes and content of physiotherapy consultations to aid communication. Brief 10 min interviews with control midwives (n=12) reported a range of previous PFME training experience, from little or no training to two with extensive knowledge about PFME. These interviews confirmed a lack of consistency in implementing PFME advice into standard care. Control midwives reported low confidence and challenges of teaching PFME and no indication of using APPEAL resources.

One hundred twenty-three women consented to be approached to take part in a post-trial interview. Aiming for 30 and sampling across the clusters, interviews were conducted with 29 women (intervention n=13, control n=16) taking 30 minutes on average. Findings showed that understanding why and how to do PFME was highly important to women. Some women in the intervention group clearly mentioned receiving APPEAL-specific resources and advice. Some aspects of PFME advice were provided to a few women in the control group as part of standard care. A consistent finding was acknowledgement of the importance of understanding why and how to do PFME although remembering to do PFME was problematic.

## Discussion

Our previous research within the APPEAL programme found PFME support for pregnant women is insufficiently provided by UK midwives.^9 10^ This feasibility and pilot cluster RCT showed that the intervention we developed could address this. Based on women who responded to the questionnaire, findings indicated that more women in the intervention clusters said their midwife had explained how to do PFME than among controls, and the confidence intervals for these results did not overlap. This ‘how-to-do’ was a critical intention of the intervention since the earlier ethnographic study indicated assumptions were often made that women knew how to do PFME.^10^ A higher proportion of women in the intervention clusters reported positive antenatal PFME practice than controls and fewer reported UI, although, as is common in feasibility and pilot trials, for both outcomes confidence intervals were wide. The numerous trial process outcomes were generally consistent with the quantitative findings.

An important strength of this study is consistency in evaluation findings. In addition, it is the only trial that we have found in the literature in which midwives have delivered PFME support rather than specialist health professionals. Provision of intervention training during the trial was affected by COVID-19: training of midwives had to be online rather than in person. We do not know whether being online was a limitation because there were some advantages: it meant no travelling time for midwives and it would make intervention training more sustainable in the future; our training fidelity checks indicated it was delivered as intended. However, there was a different COVID-19 limitation, as research midwives employed on the trial were not allowed to visit midwifery team meetings in-person to discuss any difficulties or concerns with intervention processes; had this been permitted it might have enhanced how midwives sustained PFME support for women throughout their pregnancy. Although this may have diluted trial findings it likely reflects what would happen in a non-research context, suggesting future wider implementation will be feasible.

The low questionnaire return rate from the sampled women in the trial was a limitation and lower than the 33% return rate when piloting the questionnaire. However, the return rates and characteristics of women were similar in both trial arms. Because the APPEAL intervention became part of standard care in the intervention midwifery teams, consent to take part in the trial was not necessary and women were only asked to consent to the use of their data when they returned the postnatal questionnaire. This meant women were reporting their outcomes blind to group allocation, a feature that has not occurred (to our knowledge) in antenatal PFME trials but is an important strength of the cluster design used for this study. However, the lack of prior consent did mean that we could not use online questionnaire methods, which potentially reduced the return rate. We did however have data to compare most characteristics of the women who returned questionnaires with the non-respondents and, apart from slightly fewer women in the non-respondent group who were White British and slightly fewer who were nulliparous, all other characteristics were reassuringly similar.

As described in the introduction, previous trials included in the Cochrane review^7^ tested PFME interventions delivered antenatally by specialist health professionals, mainly physiotherapists. In the UK, it would not be feasible for every woman to see a specialist PFME health professional during pregnancy. However, NICE recommends that standard UK antenatal care includes 10 midwifery appointments for nulliparous and seven for parous women,^17^ meaning midwives are ideally placed to teach and support all women to do PFME throughout their pregnancy. We are not aware of any other published trials evaluating a midwifery-led antenatal PFME intervention delivered to all women.

The APPEAL research programme commenced in March 2016, and the main aim of its final work package, the feasibility and pilot trial reported in this paper, was to ascertain whether a definitive cluster RCT could be conducted to test whether the APPEAL PFME intervention could reduce UI. In January 2019, however, the NHS England (NHSE) 10 year Long Term Plan was published which included a recommendation that ‘physiotherapy should be more widely available for the one in three women who experience incontinence after childbirth, with training and support for local clinicians working with antenatal and postnatal women, such as GPs and midwives’.^18^ This plan was launched in 2021 through the Perinatal Pelvic Health service in several parts of England (whole country to follow by March 2024). Consequently, the APPEAL intervention developed in this feasibility trial could not be tested in a full trial in England because any standard care control group would include midwives who had received recent PFME training. The NHSE team involved with putting this new service into practice knew about our research and we have worked with them in relation to implementation of APPEAL training for the NHSE Perinatal Pelvic Health services. The establishment of this new pelvic health service meant that further RCT testing could only be done in another country with a similar maternity care system.

In summary, this research programme has produced consistent and new data to demonstrate that appropriate training and resourcing midwives to teach and support women to undertake PFME during pregnancy is acceptable and feasible, change midwives’ behaviour, could improve women’s PFME adherence and might reduce postpartum UI. Despite some limitations in this programme of research, this represents the best available evidence on whether it is feasible to embed a midwife-led PFME intervention in standard antenatal care in England and how this can be done.

## supplementary material

10.1136/bmjopen-2024-091248 online supplemental file 1

10.1136/bmjopen-2024-091248 online supplemental file 2

10.1136/bmjopen-2024-091248 online supplemental file 3

10.1136/bmjopen-2024-091248 online supplemental file 4

10.1136/bmjopen-2024-091248 online supplemental file 5

10.1136/bmjopen-2024-091248 online supplemental file 6

10.1136/bmjopen-2024-091248 online supplemental file 7

## Data Availability

Data are available upon reasonable request.
