# Supplementary material for: Antenatal pelvic floor muscle exercise intervention led by midwives in England to reduce postnatal urinary incontinence: APPEAL feasibility and pilot randomised controlled cluster trial
Source: BMJ Open. 2025 Jan 20;15(1):e091248. doi: 10.1136/bmjopen-2024-091248 (PMC11751916; doi:10.1136/bmjopen-2024-091248)
Supplement: online supplemental file 1 [file bmjopen-15-1-s001.docx]

# APPEAL Trial Postal questionnaire Form v2.0 (06-Nov-2019)


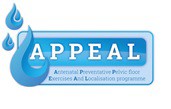


| PN Questionnaire Form |
| --- |

Unique ID number

Date of questionnaire completion: *e.g. 31-JAN-2017*

**D D** - **M M M** - **Y Y Y Y**

| Section 1 - Urinary symptoms |
| --- |

Many women leak urine following the birth of their baby some of the time. We are trying to find out how many women leak urine and how much this bothers them.

| Urinary symptoms over the past four weeks |
| --- |


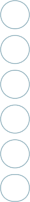

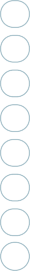
We would be grateful if you could answer the following questions, thinking about how you have been, on average, OVER THE PAST FOUR WEEKS.

| How often do you leak urine? (Tick one option) never  about once a week or less often two or three times a week about once a day  several times a day all of the time |
| --- |
| We would like to know how much urine you think leaks. How much urine do you usually leak (whether you wear protection or not)? (Tick one)  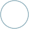 none 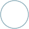 a small amount 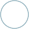 a moderate amount 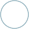 a large amount |
| Overall, how much does leaking urine interfere with your everyday life? Please tick a number between 0 (not at all) and 10 (a great deal)  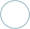 0 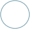 1 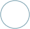 2 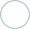 3 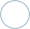 4 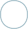 5 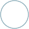 6 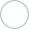 7 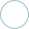 8 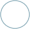 9 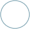 10 |
| When does urine leak? (Please tick all that apply to you) never - urine does not leak  leaks before you can get to the toilet leaks when you sneeze  leaks when you are asleep  leaks when you are physically active/exercising  leaks when you have finished urinating and are getting dressed leaks for no obvious reason  leaks all of the time |

| Urinary symptoms at the start of your pregnancy |
| --- |
| How often did you leak urine AT THE START OF YOUR PREGNANCY? (Tick one option) never  about once a week or less often two or three times a week about once a day  several times a day all of the time  can't remember |

| Section 2 - Bowel symptoms - over the past four weeks |
| --- |


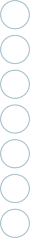

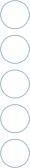
Many women have bowel symptoms following the birth of their baby some of the time. We are trying to find out how many women experience bowel symptoms and how much this bothers them. We would be grateful if you could answer the following questions, thinking about how you have been, on average, OVER THE PAST FOUR WEEKS.

| Do you leak, have accidents or lose control with solid stool? (Tick one option) never  rarely (ie. less than once in the past four weeks)  sometimes ( ie. less than once a week, but once or more in the past four weeks) often or usually (ie. less than once a day but once a week or more)  always (ie. once or more per day or whenever you have a bowel motion) |
| --- |

# ISRCTN: 37357 CONFIDENTIAL WHEN COMPLETED Page 1 of 4


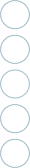

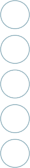

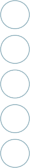

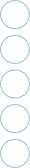

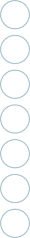


| Do you leak, have accidents or lose control with liquid stool? (Tick one option) never  rarely (ie. less than once in the past four weeks)  sometimes ( ie. less than once a week, but once or more in the past four weeks) often or usually (ie. less than once a day but once a week or more)  always (ie. once or more per day or whenever you have a bowel motion) |
| --- |
| Do you leak stool if you don't get to the toilet in time? (Tick one option) never  rarely (ie. less than once in the past four weeks)  sometimes ( ie. less than once a week, but once or more in the past four weeks) often or usually (ie. less than once a day but once a week or more)  always (ie. once or more per day or whenever you have a bowel motion) |
| Does stool leak so that you have to change your underwear? (Tick one option) never  rarely (ie. less than once in the past four weeks)  sometimes ( ie. less than once a week, but once or more in the past four weeks) often or usually (ie. less than once a day but once a week or more)  always (ie. once or more per day or whenever you have a bowel motion) |
| Does bowel or stool leakage cause you to alter your lifestyle? (Tick one option) never  rarely (ie. less than once in the past four weeks)  sometimes ( ie. less than once a week, but once or more in the past four weeks) often or usually (ie. less than once a day but once a week or more)  always (ie. once or more per day or whenever you have a bowel motion) |
| Do you leak, have accidents or lose control with gas (flatus or wind)? (Tick one option) never  rarely (ie. less than once in the past four weeks)  sometimes ( ie. less than once a week, but once or more in the past four weeks) often or usually (ie. less than once a day but once a week or more)  always (ie. once or more per day or whenever you have a bowel motion) |


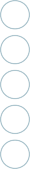
 Section 3 - Pelvic Floor Muscle Exercises


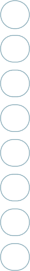
Pelvic Floor Muscle Exercises are often recommended to prevent and help with urine leakage. The exercises involve contracting (tightening and pulling up) the muscles in the area around your vagina. This section asks questions about doing Pelvic Floor Muscle Exercises.

| How often did you perform Pelvic Floor Muscle Exercises when you were pregnant? (Tick one option) never - was never advised to  never - other reasons a few times a month once a week  a few times a week once a day  a few times a day  can't remember |
| --- |
| Do you currently perform Pelvic Floor Muscle Exercises? (Tick one option) 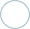 Yes 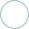 No |
| How often did you do Pelvic Floor Muscle Exercises over the last month? (Tick one option) never - was never advised to  never - other reasons a few times a month once a week  a few times a week once a day  a few times a day |


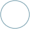

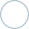

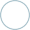

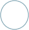

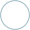

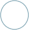

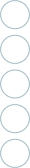

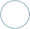

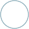

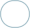

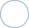

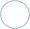

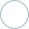

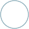

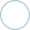

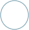

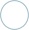

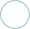

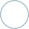

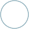

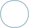

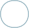

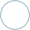

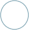

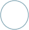

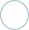

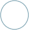

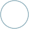

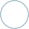

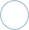

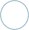

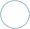

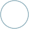

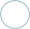

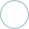

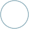


| Did your midwife advise you to perform Pelvic Floor Muscle Exercises when you were pregnant? (Tick one option) | Yes | No |
| --- | --- | --- |
| Did your midwife explain how to perform Pelvic Floor Muscle Exercises when you were pregnant? (Tick one option) | Yes | No |
| Did your midwife give you a pack of information on Pelvic Floor Muscle Exercises when you were pregnant? (Tick one option) | Yes | No |
| When did your midwife give you the pack of information on Pelvic Floor Muscle Exercises ? (Tick one option) 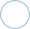 never - was never given a pack of information  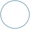 at my first (booking) appointment  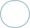 at my second midwife antenatal clinic appointment 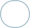 at another antenatal clinic appointment | | |
| How often did your midwife talk to you about Pelvic Floor Muscle Exercises when you were pregnant? (Tick one option) never  once only - at booking appointment occasionally  at every antenatal clinic appointment  can't remember | | |
| Did your midwife ever ask you if you had any diffi 맀怀culties with performing Pelvic Floor Muscle Exercises? (Tick one option) | Yes | No |
| Before you were pregnant have you ever been taught or learned how to perform Pelvic Floor Muscle Exercises? (Tick one option) | Yes | No |

| Section 4 - The following questions ask about confidence in relation to Pelvic Floor Muscle Exercises. | | | | |
| --- | --- | --- | --- | --- |
| I believe I can contract my pelvic floor muscles as intensive as I can (Tick one option)  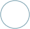 strongly disagree 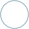 | disagree | neutral | agree | strongly agree |
| I believe I can contract my pelvic floor muscles for duration of 5 seconds (Tick one option)  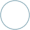 strongly disagree 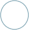 | disagree | neutral | agree | strongly agree |
| I believe I can contract my pelvic floor muscles for duration of 10 seconds (Tick one option)  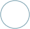 strongly disagree 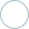 | disagree | neutral | agree | strongly agree |
| I believe I can perceive the contraction of the muscle while I am doing pelvic floor muscle exercises (Tick one option)  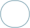 strongly disagree 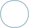 disagree 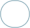 neutral | | | agree | strongly agree |
| I believe I can do pelvic floor muscle exercises while doing housework (Tick one option)  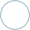 strongly disagree 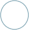 | disagree | neutral | agree | strongly agree |
| I believe I can do pelvic floor muscle exercises anytime I think of it, such as, while driving, riding or waiting for a traffi 맀怀c light change (Tick one option)  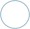 strongly disagree 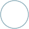 disagree 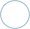 neutral 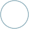 agree 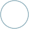 strongly agree | | | | |
| I believe I can contract my pelvic floor muscles before physical exertion, e.g, coughing, laughing (Tick one option)  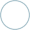 strongly disagree 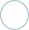 disagree 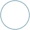 neutral | | | agree | strongly agree |
| I believe that pelvic floor muscle exercises can help decrease urine leakage (Tick one option)  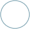 strongly disagree 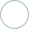 disagree 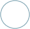 neutral | | | agree | strongly agree |
| I believe that pelvic floor muscle exercises can help avoid (or delay) incontinence surgery (Tick one option)  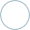 strongly disagree 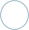 disagree 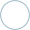 neutral | | | agree | strongly agree |
| I believe I can contract my pelvic floor muscles to increase pleasure during sexual intercourse (Tick one option)  strongly disagree disagree neutral | | | agree | strongly agree |


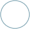

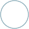

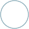

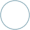

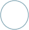

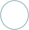


| I believe I can do pelvic floor muscle exercises even without the assistance of biofeedback and or electrical stimulation (Tick one option)  strongly disagree disagree neutral agree strongly agree | | | | |
| --- | --- | --- | --- | --- |
| I believe I can do pelvic floor muscle exercises daily (Tick one option)  strongly disagree | disagree | neutral | agree | strongly agree |
| I believe I can do pelvic floor muscle exercises regularly for 3 months (Tick one option)  strongly disagree | disagree | neutral | agree | strongly agree |
| I believe I can remind myself to do pelvic floor muscle exercises every day (Tick one option)  strongly disagree | disagree | neutral | agree | strongly agree |
| I believe I can do pelvic floor muscle exercises even when there is a lack of time (Tick one option)  strongly disagree disagree | | neutral | agree | strongly agree |
| I believe I can do pelvic floor muscle exercises even when I lack energy (too tired) (Tick one option)  strongly disagree disagree | | neutral | agree | strongly agree |
| I believe I can do pelvic floor muscle exercises while watching TV (Tick one option)  strongly disagree | disagree | neutral | agree | strongly agree |

| Section 5 - The following questions ask about how often you manage to do your pelvic floor muscle exercises | | | | | |
| --- | --- | --- | --- | --- | --- |
| I do my exercises as often as recommended (Tick one option) | strongly disagree | disagree | neutral | agree | strongly agree |
| I don't get around to doing my exercises (Tick one option) | strongly disagree | disagree | neutral | agree | strongly agree |
| I do most, or all of my exercises (Tick one option) | strongly disagree | disagree | neutral | agree | strongly agree |
| I do less exercise than recommended by my healthcare professional (Tick one option)  strongly disagree | | disagree | neutral | agree | strongly agree |
| I fit my exercises into my regular routine (Tick one option) | strongly disagree | disagree | neutral | agree | strongly agree |
| I forget to do my exercises (Tick one option) | strongly disagree | disagree | neutral | agree | strongly agree |

| Section 6 - Further Information | | |
| --- | --- | --- |
| May we contact you in the future for further research in this area? (Tick one option) | Yes | No |
| May we access your hospital maternity notes for any further relevant information (eg, type of birth)? (Tick one option) | Yes | No |
| May we contact you about speaking with a researcher about your experiences of performing pelvic floor muscle exercises in pregnancy? If YES, please provide a contact number/email address………………………………………………... | Yes | No |
| Would you like to be notified of the results of the study? (Tick one option) | Yes | No |
| Thank you very much for your help  Please return the completed questionnaire back to us in the postage paid envelope provided. | | |
