# Supplementary material for: Antenatal pelvic floor muscle exercise intervention led by midwives in England to reduce postnatal urinary incontinence: APPEAL feasibility and pilot randomised controlled cluster trial
Source: BMJ Open. 2025 Jan 20;15(1):e091248. doi: 10.1136/bmjopen-2024-091248 (PMC11751916; doi:10.1136/bmjopen-2024-091248)
Supplement: online supplemental file 2 [file bmjopen-15-1-s002.docx]

**Topic Guide for ONE MONTH post training telephone interview –**

**Midwives and Midwife Champions.**

**Introduction, thank you, any questions. Expect interview time 20 mins.**

**ALL PARTICIPANTS: Background information**

We would like to ask some questions about your experience as a midwife.

1. What year did you qualify as a midwife?

2. What is your current role?

3. Had you received any previous training/qualifications (formal or informal) relating to pelvic floor muscle exercise before this study?

**MIDWIVES AND CHAMPIONS: Experiences and views of the training session**

Explain that I would like to begin by asking about the 2 hour training session.

1 Is there anything about the training session that recall that you would like to comment on?

Prompts - What did you find particularly useful about the training?

2 Can you tell me about any part of the training that could be improved or changed?

Prompts - delivery, content

**MIDWIVES: Experiences and views regarding the one month ‘test’ period**

1 How did you find putting the training into practice (delivering the intervention?)

2 What was your experience of the women’s response to the APPEAL intervention?

- Prompt – did they provide any feedback about the pack

- How did the women respond to the verbal description of PFME

- did women report any barriers/problems to performing PFME – what helped/could help them to remember

3 How did you find the resources that were available to you as a midwife to help you deliver the training to women?

4 Is there anything else you would like to talk about related to the APPEAL training or the delivery of the APPEAL training to antenatal women?

OR

**CHAMPIONS: Experiences during the one month ‘test’ period**

1. You identified yourself as being willing to take on the role of a midwife Champion for the APPEAL study. How is that role going?
2. What has this role involved

Prompt – things that have gone well, not gone well

1. Have you had Champion training yet? If so, how was the Champion training?
2. Any other comments
