# Supplementary material for: Antenatal pelvic floor muscle exercise intervention led by midwives in England to reduce postnatal urinary incontinence: APPEAL feasibility and pilot randomised controlled cluster trial
Source: BMJ Open. 2025 Jan 20;15(1):e091248. doi: 10.1136/bmjopen-2024-091248 (PMC11751916; doi:10.1136/bmjopen-2024-091248)
Supplement: online supplemental file 4 [file bmjopen-15-1-s004.docx]

**Interview guide – Women (service-user) participants from intervention group - postnatal interview**

**This guide is indicative only and follows on from the participant information check and audio-recorded consent completion. Questions will not necessarily be asked in this order.**

| Topic | Exemplar questions |
| --- | --- |
| Background | Thank you for agreeing to take part in this research.  This study is about PFME.  We would like to talk to you about your thoughts and experiences about PFME during your pregnancy.  Before we start talking about pelvic floor exercises, please could you confirm what is your year of birth?  Please can you tell me about your recent pregnancy   - Was this your first pregnancy? - What are the ages of your other children? |
| Pelvic floor knowledge and information | Were there opportunities for you to ask questions about PF ‘health’ when you were pregnant? (e.g. what are normal changes in pregnancy, any issues that are common but not normal such as leaking, pain etc)  What advice, if any, was given about PFME by your midwife?  If given advice:   - Can you tell me what the advice/information was? - How useful was the information? - Do you feel you benefited from the information? In what way? - Would you like to have received more information about this? - Did you have any concerns about the information you received?   What, if anything, were you given by your midwife to help you with PFME? (Prompt: APPEAL resource bag, APPEAL water droplet logo – see Participant Information Sheet for example).  If given APPEAL resource:   - Do you remember when your midwife gave you the resource bag? - What did you think of the resources? (Bag, app card, video clips, leaflet?) - What was helpful or unhelpful about the resources? - What do you think could have improved the resources?   What, if any, other sources of information were you given/ did you look up about PFME?  Prompts: source/type of info, from whom (formal v informal channels, friends, family, HCPs online, where, when?)  Were you taught about PFME by any other professionals and if so who/ what advice did they give you?  Did you know anything about PFME before being given information by your midwife/ before this pregnancy?     - (if relevant) Were you given information about PFME in any previous pregnancies? What are the ages of your other children?   How could information/advice/teaching of PFME have been improved (timing, delivery, content, media)?  What do you feel is the best way for women to be given information about the pelvic floor? |
| Doing PFME | Can you remember if your midwife taught you how to perform PFME during your pregnancy?  If yes:   - Were you told why to do PFME when you are pregnant? - How were you told to do them? - Can you recall how many exercises you were advised to do? - And how often were you asked to do them?   How often, if at all, were you reminded to do PFME when you were pregnant?  Were you told who to ask if you had any questions or concerns about your pelvic floor?  Did you manage to do PFME when you were pregnant?   - How easy was it to do? - When, how often, where? - Any thoughts on how often you did them? - What do you think made it easy/difficult to do the exercises? - Is there anything that you think would have made you more likely to do your PFME?   Are you managing to do PFME now that you have had your baby?   - How easy is it to do PFME now? - How often are you managing to do them? - Has anyone reminded you to do your PFME since you had the baby? - Is there anything that would help you to do them? - Since having your baby, do you know who to ask questions about PFME?   If successfully doing PFME, what is it about you do you think that means you have done these exercises/done them routinely?  What are some of the things that may have stopped you doing PFME?  What advice would you give us about helping women to do PFME?  What would you say to a friend who asked you for advice about the pelvic floor/PFME?  What do you feel are the benefits, if any, of doing PFME (in pregnancy/post-natal)?  Do you have any concerns about PFME? |
| Pelvic floor muscle contraction technique | How confident are you that you have been doing a pelvic floor muscle contraction correctly?  Why do you think that you are/are not doing it correctly?  Has your midwife or other HCP ever asked whether you think you are doing a pelvic floor contraction correctly?   - Or offered any kind of assessment/to check? - Have you done any kind of self-assessment? - Would you have liked to have been offered the opportunity to have an assessment?   Having had your baby do you feel any differently about any of this? |
| Knowledge about pelvic floor and bladder control | One of the benefits of PFME is to help prevent and manage UI.  What have you heard about urinary incontinence (leaking urine)?  What have you heard about the link between doing PFME and incontinence?   - Where did you hear this information? - Was this something you discussed with your midwife? - Were you asked about leaking urine by your midwife? - Do you recall how often you were asked about UI? - Have any other HCP (e.g., Physios, GP?) spoken to you about UI?   Have you had any experience of this yourself?   - Can you tell me a bit more about your experiences (friends’ experiences?) - Did you feel able to tell your midwife about this? - What advice were you given? |
| Concluding | Is there anything else that you think I should have asked you about this?  Do you have anything to add?  Check participant knows where to seek further advice if required (refer to Participant Information Sheet)  Thank participant for taking part. |
