# Supplementary material for: Antenatal pelvic floor muscle exercise intervention led by midwives in England to reduce postnatal urinary incontinence: APPEAL feasibility and pilot randomised controlled cluster trial
Source: BMJ Open. 2025 Jan 20;15(1):e091248. doi: 10.1136/bmjopen-2024-091248 (PMC11751916; doi:10.1136/bmjopen-2024-091248)
Supplement: online supplemental file 5 [file bmjopen-15-1-s005.docx]

**Topic Guide for POST STUDY telephone interview – Midwives (control group)**

Introduction, thank you, any questions.

Expect the interview to take around 10 minutes to complete

**Pelvic Floor Muscle Exercises (PFME) / urinary incontinence**

1. Please will you tell me briefly what you usually say to women during their ante-natal care regarding PFME (or about preventing urinary incontinence)

*Prompt:* do you usually manage to mention it at the booking appointment / other appointments…..how do you find fitting in this information / advice……

*Prompt:* have you had any training regarding PFME? If so what did this consist of….

1. Have you changed the way you practice recently (regarding advice you give about PFME or urinary incontinence)

*Prompt:* please describe the change…..please say why you decided to make these changes…..did you hear about the APPEAL study……

**The APPEAL study**

You were asked to let women know about a questionnaire being sent to them after their baby was born. The APPEAL questionnaire was about whether they did pelvic floor muscle exercises and assessing whether they helped to prevent urinary incontinence during or after pregnancy.

1. Is there anything about the APPEAL study that you would like to comment on?
2. When did you first hear about the APPEAL study?

*Prompt:* What did you hear about APPEAL? Do you have any views about being in the usual care arm of the study?

1. Did hearing about the study change the way you practiced in any way…..

*Prompt:* If yes, in what way…..when…..did you continue with these changes…

1. Now, at the end of the study, is there anything that you can recall about having to let women know about the APPEAL questionnaire….

*Prompt:* What was it like to tell the women about the questionnaire? ….or the topics it contained? ….were there any issues with doing this?

1. Do you have any other feedback about the APPEAL study?

*Prompts* (**only if not already covered**) - can I please check did you get to hear about APPEAL before you were told about the questionnaires? What did you hear about APPEAL? Did hearing about the study change the way you practiced in any way…..

1. Is there anything else you would like to talk about related to APPEAL or the delivery of PFME advice to antenatal women?

*Prompt:* how do you feel about having to fit in extra PFME advice and support during ante-natal appointments?

1. Any other comments?
