# Supplementary material for: Antenatal pelvic floor muscle exercise intervention led by midwives in England to reduce postnatal urinary incontinence: APPEAL feasibility and pilot randomised controlled cluster trial
Source: BMJ Open. 2025 Jan 20;15(1):e091248. doi: 10.1136/bmjopen-2024-091248 (PMC11751916; doi:10.1136/bmjopen-2024-091248)
Supplement: online supplemental file 6 [file bmjopen-15-1-s006.docx]

**Topic Guide for END OF STUDY telephone interview –**

**Midwives and Midwife Champions**

Introduction, thank you, any questions. Expect the interview to take around 20 minutes to complete

**MIDWIVES AND CHAMPIONS: The training**

1 Is there anything about the training that you can recall that you would like to comment on?

Prompts - Now, at the end of the study, is there anything that you can recall about the training that you found particularly valuable?

Are there any improvements or changes that you would find helpful?

- delivery, content

2 Do you have any other feedback about the training session?

Prompts - refresher courses – format and content?

**MIDWIVES: Intervention delivery over the antenatal period**

1. What resources are, or would be required if this intervention was to be rolled out or implemented in the future (prompt – referral rates, service level input)
2. Having given out the resource pack, how did the progression checks go at each antenatal appointment

- Prompt - How did the women respond to the verbal description of PFME

1. How did you find the resources that were available to you as a midwife to help you deliver the training to women

- Prompt – how did you find the role of the midwife champion within the team?

1. Is there anything else you would like to talk about related to the APPEAL training or the delivery of the APPEAL training to antenatal women

***OR***

**CHAMPIONS: Experiences of your role as APPEAL Champion**

1. You identified yourself as being willing to take on the role of a midwife Champion for the APPEAL study. Can you tell me about what this role involved

Prompt – things that have gone well, not gone well, resources required

1. Any other comments
