# Supplementary material for: Antenatal pelvic floor muscle exercise intervention led by midwives in England to reduce postnatal urinary incontinence: APPEAL feasibility and pilot randomised controlled cluster trial
Source: BMJ Open. 2025 Jan 20;15(1):e091248. doi: 10.1136/bmjopen-2024-091248 (PMC11751916; doi:10.1136/bmjopen-2024-091248)
Supplement: online supplemental file 7 [file bmjopen-15-1-s007.docx]

**APPEAL Questionnaire**

We would like to find out how you have got on with the APPEAL intervention over the last few months. This is a feasibility trial so we need your **honest feedback** as this will really help us to understand more about the APPEAL intervention in a real-life clinical setting. Your responses are anonymous.

The APPEAL intervention has several components. We would like to know which, if any, you are able to incorporate into your routine care.

Some questions may not be applicable to you, depending on the nature of your role.

I am a *(please circle)* MW MSW Other……………………….……….

**Please circle your responses.**

**1.      Do you raise the topic of pelvic floor muscle exercises (PFME) with women the first time you see her antenatally?**

Yes, all women / Yes, most women / Yes, some women/ Yes, a few women / No / Not Applicable

**2.    Have the women under your care been given the APPEAL resource bag?**

 Yes, all women / Yes, most women / Yes, some women/ Yes, a few women / No women

**3.    Do you discuss the contents of the APPEAL resource bag as you give them out?**

  Yes, all women / Yes, most women / Yes, some women/ Yes, a few women / I don’t discuss it

**4.    How often do you ask women about UI?**

At all appointments / At most appointments/ At some appointments / Never

**5. Are you managing to teach women how to do their pelvic floor muscle (PFM) contraction?**

Yes, all women / Yes, most women / Yes, some women/ Yes, a few women / No women

**6.** **Are you managing to get women to practise a PFM contraction during an antenatal appointment with you?**

Yes, all women / Yes, most women / Yes, some women/ Yes, a few women /No women

**We would welcome any further comments on this** ……………………………………………………………………………………………………………………………………………………………………………………………………………………………………………………………………………………………………………………………………………………………………………………………………………………………………………..

**7. Are you managing to agree and set an individualised PFM exercise programme with women to practise at home?**

yes, All women / yes, Most women / yes, Some women/yes, A few women / No women

**8.  Are you managing to help women review their progress with their PFM exercise programme throughout pregnancy? (eg increasing from four second holds to six second holds)**

Yes, all women / Yes, most women / Yes, some women/ Yes, a few women / no women

**9. Have you needed to refer women to the physiotherapy department for managing incontinence?**

Yes If yes, how many? ……………… No

**10. Are there any barriers to you implementing the APPEAL intervention and if so, what? (tick all that apply)**

- Not mandatory
- Language barriers
- Not on Badgernet
- Other (please add) …………………………………………….
- No barriers
- Lack of time
- Forgetting
- Other priorities
- Not my role

**11. Is there anything that helps you to implement the APPEAL intervention? (tick all that apply)**

- Team champion
- APPEAL prompt card
- Confidence with training
- Knowing how to refer
- Other (please add) ……………………………………………….
- Community team support
- APPEAL research team support
- APPEAL training folder
- APPEAL women’s resource bag

**12. Any other comments?**

………………………………………………………………………………………………………………………………………………………………………………………………………………………………………………………………………………………………………………………………………………………………………………………………………………………………………………………………………………………………………………………………………………………………………………………………
