# Supplementary material for: Antenatal pelvic floor muscle exercise intervention led by midwives in England to reduce postnatal urinary incontinence: APPEAL feasibility and pilot randomised controlled cluster trial
Source: BMJ Open. 2025 Jan 20;15(1):e091248. doi: 10.1136/bmjopen-2024-091248 (PMC11751916; doi:10.1136/bmjopen-2024-091248)
Supplement: Supplementary file 8 [file bmjopen-2024-091248.reviewer_comments.pdf]

PEER REVIEW HISTORY

BMJ Open publishes all reviews undertaken for accepted manuscripts. Reviewers are asked to complete a checklist review form and are provided with free text boxes to elaborate on their assessment. These free text comments are reproduced below.

ARTICLE DETAILS

Title (Provisional)

Antenatal pelvic floor muscle exercise intervention led by midwives in England to reduce postnatal urinary incontinence: APPEAL feasibility and pilot randomised controlled cluster trial

Authors

MacArthur, Christine; Bick, Debra; Salmon , Victoria; Jones, Ellie; Hay-smith, Jean; Bishop, Jonathan; Gkini, Eleni; Hemming, Karla; Webb, Sara; Pearson, Mark; Coleman, Tim; Terry, Rohini H; Edwards, Elizabeth; Frawley, Helena Catherine; Oborn, Eivor; Dean, Sarah Gerard

---

VERSION 1 - REVIEW

**Reviewer**	**1**
**Name**	**Ruseckaite, Rasa**
**Affiliation**	**Monash University, Public Health and Preventive Medicine**
**Date**	**11-Sep-2024**
**COI**	**None**

Thank you for the opportunity to review this manuscript. The aim of this study was to assess feasibility of an intervention of midwifery support for antenatal pelvic floor

muscle exercises (PFME) to prevent postnatal urinary incontinence. This is an interesting piece of work, however I would like to recommend some improvements"

1. Please define "feasibility" in your study.

2. Methods section would benefit from adding a sub-headings to describe your study population, intervention, clusters, survey and how it was designed, qualitative component, data analyses and etc.

3. Please provide more details about your questionnaire and questions asked. I understand, it might have been described separately in the protocol, but it would be great if your reader did not need to look for it and could access some information in this manuscript.

4. It appears that this was a mixed methods study - qualitative component should be also described.

5. Please describe how the returned survey data were stored, was it cleaned for QA purposes?

6. Please provide more details about the interview questions, how the topic guide was designed, etc.

7. I am not so sure whether it is important to report every single ethnicity in your Table 1, especially when your numbers and response rates are so low. Could some of these be aggregated? I would also suggest omitting "missing" data from your tables.

8. Response rates of your survey were very low. This is a major limitation in your study and needs to be addressed in a greater detail. Was this expected, and if you had any plans to mitigate this particular risk? Would it be possible to repeat this study to achieve the required sample size and to strengthen your findings and conclusions?

**Reviewer**	**2**
**Name**	**Huebner, Markus**
**Affiliation**	**University of Freiburg, Hugstetter**
**Date**	**22-Sep-2024**
**COI**	**none**

Title: Antenatal pelvic floor muscle exercise intervention led by midwives to reduce postnatal urinary incontinence: APPEAL feasibility and pilot randomised controlled cluster trial

Introduction Summary:

Novelty: given

Methodology: well written

Presentation: well written

Population: community maternity antenatal care, 175 women, 186 midwives.

Study Design: feasibility and pilot cluster randomized controlled trial

Inclusion Criteria: women who gave birth during December 2021

Exclusion Criteria: stillbirth, neonatal death

Primary outcome: women reporting: the midwife explained how to do PFME; PFME appearance; postpartum UI. Midwives reporting: pre-post-training PFME confidence; intervention acceptability, Fidelity of training delivery and implemntation

Statistics: intention-to-treat-principle, descriptive

Results: 95/91 midwives, 15-8% and 16.4% responded. 65% of women in intervention clusters reported their midwife explained ho to do PFME vs 38% in control clusters. 50% vs.38% reported doing PFMT. Intervention training was delivered with fidelity and received positively. Improvement in PFME confidence/knowledge. Implementation barrier: insufficient time.

Conclusions: training and resourcing midwives to teach/support pregnant women to undertake PFME is acceptable and feasible for both women and midwives. Increased the numerb of women who are informed about PFME with potential to improve PFME adherence and reduce postpartum UI.

Questions/comments for the Authors:

The article "Antenatal pelvic floor muscle exercise intervention led by midwives to reduce postnatal urinary incontinence: APPEAL feasibility and pilot randomised controlled cluster trial" is an interesting manuscript. I do not have anything to add. Congratulations.

VERSION 1 - AUTHOR RESPONSE

**Reviewer 1:**	**Response to reviewers**
1. Please define "feasibility" in your study.	The following text has been added on page 4:<br><br>'In a randomized controlled trial (RCT), feasibility refers to the process of evaluating whether a trial is possible to conduct and if all the necessary components will work together. The goal of a feasibility study is to determine if a study can be done, if it should be done, and how it should be done'.
2. Methods section would benefit from adding a sub-heading to describe your study population, intervention, clusters, survey and how it was designed, qualitative component, data analyses and etc.	Subheadings '*study population*', '*intervention*', '*cluster*' and '*survey*' have been added on pages 4-7.
3. Please provide more details about your questionnaire and questions asked. I understand, it might have been described separately in the protocol, but it would be great if your reader did not need to look for it and could access some information in this manuscript.	The women's postal questionnaire and interview topic guides have now been added as supplementary material 1-6.
4. It appears that this was a mixed methods study - qualitative component should be also described.	We believe this is already described under process evaluation on page 6

5. Please describe how the returned survey data were stored, was it cleaned for QA purposes?	The following text has been added on page 5:<br><br>*'Questionnaires were returned to participating NHS Trusts and transferred securely in line with standard data protection procedures to Birmingham Clinical Trials Unit (BCTU) for data cleaning and analysis by BCTU statisticians'.*
6. Please provide more details about the interview questions, how the topic guide was designed, etc.	The following text has been added on page 6-7:<br><br>*'Topic guides (supplementary material 2-6) for the various midwives' and women's interviews were developed based on previous work packages including: critical interpretive synthesis (11), focussed ethnographic observation of clinical practice (10) and input from Patient and Public Involvement and Engagement (PPIE) advisory groups'.*
7. I am not so sure whether it is important to report every single ethnicity in your Table 1, especially when your numbers and response rates are so low. Could some of these be aggregated? I would also suggest omitting "missing" data from your tables.	The senior statistician is very keen to keep missing values in table 1 and not to collapse ethnic groups.
8. Response rates of your survey were very low. This is a major limitation in your study and needs to be addressed in a greater detail. Was this expected, and if you had any plans to mitigate this particular risk? Would it be possible to repeat this study to achieve the required sample size and to strengthen your findings and conclusions?	The following text has been added in the discussion on page 11:<br><br>'*The low questionnaire return rate from the sampled women in the trial was a limitation and lower than the 33% return rate when piloting the questionnaire'.*<br><br>And in addition the following on page 12:<br><br>*The NHSE team involved with putting this new service into practice knew about our research and we have worked with them in relation to implementation of APPEAL training for the NHSE Perinatal Pelvic Health services*<br><br>We have now added:<br><br>*'The establishment of this new pelvic health service meant that further RCT testing could therefore only be done in another country with a similar maternity care system'.*
